# Understanding Measles in Hospitalized Adults: Insights into the Recent Romanian Epidemic, Clinical Presentation and Perspectives on MMR Vaccination Among Roma Hospitalized Patients

**DOI:** 10.3390/vaccines14070612

**Published:** 2026-07-14

**Authors:** David Valentin Mangaloiu, Dag S. Halvorsen, Alexandra Denisa Raris, Aramă Sorin Ștefan, Victoria Aramă, Florentina Ligia Furtunescu

**Affiliations:** 1 Doctoral School, “Carol Davila” University of Medicine and Pharmacy, 020021 Bucharest, Romania; sorinarama@gmail.com (A.S.Ș.); dr.arama@mateibals.ro (V.A.); florentina.furtunescu@umfcd.ro (F.L.F.); 2 Faculty of General Medicine, Discipline of Public Health and Management, “Carol Davila” University of Medicine and Pharmacy, 020021 Bucharest, Romania; 3 Department of Clinical Medicine, UiT The Arctic University of North Norway, 9037 Tromsø, Norway; dag.s.halvorsen@uit.no; 4 Department Pediatrics, Emergency Clinical Hospital for Children “Marie S. Curie”, 400015 Bucharest, Romania; alexandra-denisa.raris@rez.umfcd.ro; 5 “Prof. Dr. Matei Bals” National Institute for Infectious Diseases, 1 Calistrat Grozovici Street, 021105 Bucharest, Romania

**Keywords:** measles outbreak, Roma ethnicity, vaccine hesitancy, adults

## Abstract

Background/Objectives: Measles remains a significant public health concern in Romania, with recurrent ongoing nationwide outbreaks despite the availability of the measles–mumps–rubella (MMR) vaccine. This study investigates the epidemiological, clinical, and sociocultural dimensions of measles among Romanian adults, with a particular focus on a vulnerable group, the Roma population. Methods: We conducted a retrospective cohort study using clinical data from a tertiary hospital in Bucharest, Romania. The study included adult patients hospitalized with measles between July 2023 and April 2024. In a subsequent phase, we carried out a cross-sectional survey among hospitalized measles patients to assess their perception and understanding of measles and the MMR vaccine, with particular attention to responses from Roma participants. Results: A retrospective investigation of 100 hospitalized adult patients with laboratory-confirmed diagnoses of measles demonstrated frequent complications such as hepatic involvement (85/100), pneumonia (68/100), and respiratory failure (21/100). Only 6/100 of patients were fully vaccinated. Rhabdomyolysis was significantly more common in unvaccinated individuals and women. No deaths were recorded, and no ICU admissions occurred. Among the hospitalized patients, 49 adults responded to a vaccine centered questionnaire. We report a notable vaccine hesitancy, particularly among the Roma respondents. Socioeconomic factors such as low income, limited education, and lack of health insurance were significantly associated with negative perceptions of the MMR vaccine. A statistically significant association was observed between Roma ethnicity and the belief that the MMR vaccine causes autism. Conclusions: These findings highlight the urgent need for targeted public health interventions with culturally adapted education campaigns to improve vaccination coverage and thereby protect vulnerable adult populations in Romania.

## 1. Introduction

Measles is a highly contagious and vaccine preventable viral disease. Despite the availability of a safe and effective vaccine, many European countries experience outbreaks and epidemics with high morbidity and mortality rates [1]. In 2024, the World Health Organization (WHO) European region reported the highest numbers of measles cases since 1997, and Romania and Kazakhstan were the two countries most heavily affected [2]. Often considered a childhood disease, measles can infect individuals of all ages, with risk of complications such as pneumonia, encephalitis, hepatic dysfunction and death [3].

Romania introduced a single-dose vaccine for infants in 1979, adding a second dose for older children in 2005. After adjustments of the national vaccine strategy, the program now consists of the MMR (measles–mumps–rubella) vaccine given at 12 months and 5 years of age, provided cost-free at local health centers [4,5].

Romania has experienced recurrent outbreaks of measles during recent decades, reporting more than 20.000 cases and 64 deaths during the 2016–2020 epidemic [6,7]. Despite national efforts to improve vaccination rates, the Ministry of health declared another ongoing epidemic from December 2023, and by the end of 2025, more than 35.736 cases and more than 30 deaths were registered [8,9].

Surveillance data from the recent epidemic indicate a high number of cases in unvaccinated or incompletely vaccinated individuals, particularly affecting vulnerable groups and marginalized communities [6,9].

In Romania, one of the most vulnerable population groups is the Roma community. This ethnic group faces a disproportionate burden of disease due to a series of socioeconomic factors including poverty, overcrowding, and limited education and access to healthcare [10]. The historical Roma experience of discrimination can foster distrust of health authorities and the healthcare system [11]. Altogether, these factors contribute to reduced health-care-seeking behavior, vaccine hesitancy and poor vaccine coverage [12].

Childhood vaccination is crucial for preventing measles, but adult immunity also plays an important role in controlling outbreaks. Adults with inadequate immunity due to non-vaccination, non-exposure or immunosuppression are susceptible to measles and pose a risk for further transmission to non-immune individuals. Ensuring high levels of adult immunity through vaccination is essential for maintaining herd immunity. The health status of Roma adults has a major impact on the health status of the younger generations [13,14,15].

The purpose of the study was to analyze the characteristics of measles in adults, with a focus on the specific features observed among Roma patients by gaining insights into (1) the clinical presentations, complications and outcomes in hospitalized adults and (2) the knowledge, attitudes and perceptions of the MMR vaccination among hospitalized Roma and non-Roma patients.

## 2. Materials and Methods

### 2.1. Study Design, Settings and Population

This study explored aspects contributing to the recent measles epidemic in Romania by focusing on the adult population, analyzing data from multiple sources to provide further understanding of the epidemic situation.

For this purpose, we conducted a retrospective observational study including adult patients hospitalized with measles at the National Institute of Infectious Disease “Prof. Dr. Matei Bals (INBIMB), Department 3, which is a tertiary teaching hospital in Bucharest, Romania, where only adult patients with infectious diseases are treated and exceptionally adolescents over 14 years. During the study period from July 2023 to April 2024, there were 116 individuals admitted with measles. Sixteen patients were excluded due to younger age, short hospitalization (<48 h) or incomplete data. Inclusion criteria were represented by patients aged over 18 years with a confirmed measles diagnosis via a Real Time Polymerase Chain Reaction test (RT-PCR) or serology. Viral genotyping/whole-genome sequencing was not performed on this cohort. We excluded patients who did not require continuous hospitalization (>48 h), patients aged between 14 and 18 years old, or those with insufficient clinical or laboratory data for analysis.

All patients included were evaluated clinically, biologically and radiologically within the first hour of admission. The diagnosis was confirmed with PCR analysis from throat swabs or serology, which was collected at least 3 days after rash onset. Regarding specific detection of RNA by an RT-PCR, this technique was used to confirm suspected cases. Both serological and nasopharyngeal swabs were collected in the ward and analyzed at the Romanian National Institute of Public Health (INSP) reference laboratory.

The clinical diagnosis was based on presence of fever; generalized rash; and at least one of cough, coryza, or conjunctivitis, according to the case definition provided by WHO, European Center for Disease Control (ECDC) [1,3]. The study focused on socio-demographic and epidemiological background data, clinical presentation, complications and outcomes among hospitalized adults with measles. Data on ethnicity were not collected in the patient medical records.

Another important component of our study is a second investigation, designed as a cross-sectional sub-analysis within the initial cohort, which included 49 respondents out of 100 patients analyzed in the first study. A questionnaire that consisted of 16 items was applied to find out participants’ perceptions and understanding of measles and the MMR vaccine, with a specific focus on Roma individuals. The covering areas of the questionnaire were attitudes, beliefs and knowledge of the MMR vaccine and socio-demographic aspects. The questionnaire was completed through a face-to-face interview by the study author. Roma affiliation was established by their response to a specific question about ethnicity. All patients gave written consent for study participation after being given thorough information.

Data provided by the Romanian National Institute of Public Health through the national measles surveillance program was used to contextualize the discussion and to offer an overview of demographic and epidemiological aspects of the current outbreak. Information available from the national 2023–2025 measles epidemic 2025 was analyzed to provide better understanding of the outbreak’s dynamics [8]. In particular, the roles of age and vaccination were examined for discussions. The national data analysis did not account for ethnicity, as this variable is not collected.

### 2.2. Ethical Considerations

The study protocol was reviewed and approved by the Ethics Committee of the National Institute of Infectious Diseases “Prof. Dr. Matei Bals” (Approval No. 003576/05.04.2024). All research activities conformed to relevant ethical guidelines and regulations, including the Declaration of Helsinki, and written informed consent was obtained from all participants.

Regarding the information about the national outbreak of measles available on the INSP website, no written consent was required for their analysis, as these data are published monthly on the institution website and are publicly accessible to all interested individuals.

### 2.3. Statistical Analysis

Statistical analysis was performed using Statistical Package for the Social Sciences (SPSS) software, version 26. Continuous variables were summarized using the mean, median and standard deviation, whereas categorical variables were described using percentages/proportions. To assess differences between groups, the Chi-square test was used for categorical data and the Mann–Whitney test for continuous data. Univariate analysis was conducted using the independent t-test for continuous variables and Pearson’s Chi-square test for categorical variables. Fisher’s exact test was employed for the analysis of contingency tables involving categorical variables. All statistical tests were two-tailed, and a *p*-value less than 0.05 was interpreted as indicating statistical significance. A multivariate regression analysis was not conducted due to the limited sample size of Roma respondents. We used Firth penalized logistic regression to address the small-sample bias and to ensure model stability given the limited number of outcome events. Firth penalized logistic regression was conducted in R (version 4.3.2; R Core Team, Vienna, Austria) using the logistf package (version 1.24).

## 3. Results

### 3.1. Characterization of Hospitalized Adult Measles Cases

From July 2023 to April 2024, 100 adults with laboratory confirmed diagnosis of measles were hospitalized in INBIMB Department 3. The patients were living in urban Bucharest and its rural surroundings. The mean age was 35 years (SD 11.32), 53/100 were females and four were pregnant at presentation. The duration of symptoms prior to admission ranged from 1 to 10 days (median 5.5 days). Almost all patients were admitted within 3 days after debut of a rash, and the patients presented typical clinical manifestations of measles (Table 1). Surprisingly, gastrointestinal symptoms like nausea, vomiting and diarrhea were common. Hypertension (10/100), diabetes mellitus (5/100), immunosuppressive conditions (11/100) and chronic respiratory illness (7/100) were reported as comorbidities. Vaccination history was incomplete among most patients, where only 6/100 had received the two-dose regimen, while 33/100 were unvaccinated and 54/100 had an unknown status.

Symptomatically, the cohort exhibited a high prevalence of characteristic clinical manifestation, including rash (100/100), fever (99/100), and conjunctivitis (56/100). Koplik spots were observed on 79/100 of patients and pharyngitis was present in 76/100. Respiratory symptoms such as cough (92/100) and runny nose (36/100) were also common, alongside gastrointestinal symptoms like nausea or vomiting (68/100) and diarrhea (25/100) (Table 1).

The laboratory findings at admission are summarized in Table 2, together with the recorded complications, treatments, and clinical outcomes. The diagnosis was established by presence of IgM antibodies in 83/100 of the cases, and in the remaining by PCR.

The thrombocyte count was significantly lower in patients with keratoconjunctivitis (*p* = 0.003). Also, the CK level was higher in patients with hepatitis (*p* = 0.004) and in women (*p* = 0.001). Fibrinogen levels were significantly lower in patients with hepatic involvement (*p* = 0.004), which may indicate consumptive coagulopathy or liver dysfunction induced by the virus. The MDW index was significantly higher in patients with acute respiratory failure (*p* = 0.034), suggesting a potential role of this marker in assessing the severity of systemic inflammation in measles patients.

Hepatic involvement was the most common complication (85/100), followed by pneumonitis (68/100) and ocular involvement (31/100). Secondary bacterial infections were identified in 29/100 cases, and respiratory failure occurred in 21/100 patients. All patients survived the infection, and the median length of the hospital stay was 5.5 days with a range of 3 to 15 days.

Hospital length was prolonged in patients with acute respiratory failure (*p* = 0.007) and keratoconjunctivitis (*p* = 0.001), suggesting a more severe prognosis. In contrast to hypertension, diabetes mellitus was more common in patients with acute respiratory failure (*p* < 0.001), suggesting an increased vulnerability to measles-related respiratory complications. We observed an association between female sex and rhabdomyolysis (*p* = 0.001), indicating a higher susceptibility of women to this complication.

Although measles is a viral illness, nearly one-third of patients received antibiotics during hospitalization, and 18/100 had been prescribed antibiotics prior to admission. The most commonly prescribed antibiotics were amoxicillin–clavulanic acid, ampicillin, azithromycin, and ceftriaxone; only two patients received broad-spectrum agents. In 33/100 cases, the treatment was targeted, with *Staphylococcus aureus*, *Streptococcus pneumoniae*, and *Haemophilus influenzae* being the most frequently isolated pathogens in culture. Corticosteroids were administered to patients presenting with respiratory failure or with extensive/severe rash.

Pregnant women had a mean age of 24.75 years. Two of them were in the second trimester and two in the third. Half of them received antibiotics, and one developed respiratory failure requiring 3 L/min of supplemental oxygen. Among those treated with antibiotics, sputum cultures were positive for *Staphylococcus aureus* and *Haemophilus influenzae*.

Complete vaccination did not prevent acute respiratory failure (*p* = 0.619) or pneumonia/pneumonitis (*p* = 0.491) in our study. However, the percentage of unvaccinated patients is high across all categories, indicating that measles primarily affects individuals without adequate vaccination history. Additionally, rhabdomyolysis is more frequent in unvaccinated patients (*p* = 0.003), suggesting a potential protective role of vaccination against this complication.

### 3.2. Perceptions of Hospitalized Adults Regarding Measles and the MMR Vaccine

Out of 100 patients admitted to our department, 49 agreed to answer a questionnaire regarding the level of knowledge, attitudes, and beliefs about the MMR vaccination during the period of hospitalization. The mean age of respondents was 32.41 (SD 12.20), with a minimum of 18 and a maximum of 55 years old. The gender distribution of the respondents is relatively balanced, with 26/49 female and 23/49 male participants Table 3.

**Table 3:** Level of knowledge, attitudes, and beliefs regarding the MMR vaccination among adults hospitalized with measles.

	General Population (n = 32)	Roma (n = 12)	*p* Value
Socio-demographic			
Age (years/mean/SD)	35.53 ± 11.68	23.75 ± 8.14	0.001
Gender, n	Male 13/32 Female 19/32	Male 5/12 Female 7/12	0.952
Education, n	Without studies 2/32 Primary/secondary school 4/32 High school 11/32 University studies 15/32	Without studies 1/12 Primary/secondary school 8/12 High school 2/12 University studies 1/12	0.008
Religion, n	Orthodox 32/32 Protestants 0/32	Orthodox 11/12 Protestants 1/12	0.099
Income, n	No income 6/32 Low income * 14/32 High income ** 12/32	No income 8/12 Low income 3/12 High income 1/12	0.045
Health insurance, n	Yes 32/32 No 0/32	Yes 6/12 No 6/12	0.000
Perception of measles severity and effectiveness of vaccination			
Do you consider measles to be a serious disease? n	Yes 29/32 No 3/32	Yes 10/12 No 2/12	0.497
I believe that vaccination prevents the occurrence of measles. n	Yes 22/32 No 10/32	Yes 10/12 No 2/12	0.333
I believe it is better for my child to build immunity through illness than vaccination. n	Yes 6/32 No 26/32	Yes 3/12 No 9/12	0.582
Attitude toward MMR vaccination and autism belief			
Were your children vaccinated according to the MS schedule? n	Yes 25/32 No 7/32	Yes 5/12 No 7/12	0.077
Did they receive the MMR vaccine? n	Yes 26/32 No 5/32 I do not know 1/32	Yes 6/12 No 6/12 I do not know 0/12	0.141
Do you believe the MMR vaccine causes autism? n	Yes 2/32 No 19/32 I do not know 11/32	Yes 7/12 No 3/12 I do not know 2/12	0.016

* Low income < 800 euro/month; ** high income > 800 euro/month; n—number; SD—standard deviation, MMR—measles–mumps–rubella vaccine.

The majority of respondents identify as “Romanian–general population” (32/49), followed by “Roma” (12/49) and a smaller percentage of “Ukrainians” (2/49). Additionally, 3/49 of participants did not specify their ethnicity. The marital status distribution shows that 27/49 of respondents are married, while 22/49 are unmarried.

The educational background of respondents varies significantly, with the largest groups having completed university studies (16/49) and high school education (16/49). A smaller proportion completed primary school (6/49), eight classes (7/49), or vocational school (1/49), while 2/49 have no formal education. This diverse educational distribution allows for an analysis of how education level influences opinions on vaccination and health-related beliefs. The vast majority of respondents identify as Orthodox (46/49), with a small percentage being Pentecostal (2/49) and Evangelical (1/49).

Regarding vaccination according to the national program, the dataset reveals a mix of opinions. While a majority of parents have vaccinated their children (31/49), there is still a significant portion who either have not done so or are uncertain (18/49). When it comes to the MMR vaccine, 15/49 parents have not vaccinated their children, and 33/49 have vaccinated them. Interestingly, some parents answered “I don’t know” when asked whether their children had received the MMR vaccine. Of those who abstained from vaccination, the primary reasons for not doing included fear of side effects and concerns about vaccine safety, belief in natural immunity being superior and the lack of registration with a family doctor.

Concerning the belief that the MMR vaccine causes autism, according to the survey data, 25/49 of respondents firmly reject this theory, recognizing that there is no credible scientific evidence to support it. However, 8/49 believe in a connection between the MMR vaccine and autism. Notably, 16/49 of respondents are uncertain, indicating a significant level of doubt or lack of knowledge regarding the issue. Bivariate analysis shows a statistically significant association between ethnicity and the belief that the MMR vaccine may cause autism. However, the association is unadjusted and the overall correlation between the two variables was weak to moderate (Pearson R = 0.255).

We analyzed whether Roma ethnicity was associated with the belief that the MMR vaccine causes autism. The results indicate a statistically significant association between these variables (*p* = 0.032), but the results should be interpreted with caution due to the small number of Roma participants.

Given the limited sample size, applying a multivariate binary logistic regression model was not feasible, as the resulting estimates for additional factors influencing the outcome would not have been sufficiently trustworthy and valid.

However, we applied Firth’s penalized logistic regression, first estimating the ethnicity effect in a univariable model and then re-estimating it in separate models that added, one at a time, the socioeconomic covariates of concern (age, low income, lack of health insurance, low education and non-employment) in order to assess whether the observed association persisted after accounting for each potential confounder. We found the association between Roma ethnicity and belief in an MMR autism link remained strong and statistically significant after separate adjustments for age (OR 5.7, 95% CI 1.27–44.5), low income (OR 4.52, 95% CI 1.37–26.32), lack of health insurance (OR 5.56, 95% CI 1.42–37.05), low education (OR 3.89, 95% CI 1.46–27.35), and non-employment (OR 6.17, 95% CI 1.12–42.93), with each of these covariates being non-significant.

Regarding acceptance of the MMR vaccine, there was a clear negative association between the number of children and acceptance. Families with one child reported a 92% acceptance rate, whereas among those with two or more children, acceptance dropped to around 50%. Firth logistic regression revealed a substantial decrease in the odds of acceptance with each subsequent kid (OR = 0.26, *p* = 0.032), and Spearman’s correlation confirmed this pattern (rho = −0.49, *p* = 0.009). After eliminating extreme values, the effect stayed constant, indicating a strong difference between families with one kid and those with two or more.

We also observed that income significantly influences adherence to the general vaccination schedule, with lower-income parents being less likely to vaccinate their children according to it (*p* = 0.033). The majority of Roma individuals have a low income or no income 11/12, compared with 20/32 of the Romanian population. We did not observe a significant impact on MMR vaccination, as the differences are not statistically significant.

The majority of respondents (34/49) believe that vaccination helps to prevent measles, while a minority (15/49) either disagrees or is unsure about its effectiveness. Among Roma adults, only 2/12 do not believe in vaccine efficacy, compared with 10/32 of Romanians. The skepticism toward vaccine effectiveness is often linked to a preference for “natural immunity,” with some respondents 10/49 believing that it is better for children to contract the disease and develop resistance. Those who believe in natural immunity tend to be more vaccine hesitant when it comes to MMR vaccination (*p* = 0.009). Individuals who view measles as a severe illness 42/49 are more likely to support vaccination.

## 4. Discussion

In this study, we analyzed data from adults hospitalized with measles, providing a comprehensive overview of their clinical characteristics, as well as their perspectives on measles and the MMR vaccine. Our data shows that measles should not be classified solely as a childhood disease. National data report that 13.50% of those confirmed with measles during this outbreak were aged more than 20 years [9,14].

In the 2024 epidemiological report, ECDC stated that there was an escalation of measles transmission within the union. Romania had the highest notification rate (1610 cases per 1,000,000) and accounted for almost 87% of all European measle cases [16]. National data indicate that the recent Romanian epidemic lasted from January 2023 until July 2025, involving more than 35,000 cases and 30 deaths. The maximum numbers peaked in March 2024 and to a lesser degree in March 2025, affirming seasonal variations [9].

Most of the Romanian cases occurred in children aged 1–4 years, but a high proportion of the infected individuals was older than 15 years of age (22.4%), and 13.50% was older than 20 years old [9].

The data align with prior evidence showing that measles is no longer viewed exclusively as a childhood disease [14]. Although in smaller numbers, Italy, Spain, France and Poland reported the most cases in adults older than 30 years [16]. This could be explained by different age-related vaccination coverages or catch-up strategies to close the immunity gaps [17].

According to the National Institute, the number of reported cases showed considerable regional variability. Counties such as Brasov, Alba, Calarasi and Giurgiu reported the highest incidences (260–383 cases per 100,000 inhabitants), while other regions were scarcely affected [9,18]. Counties with low reported incidence generally correspond to areas with higher vaccination uptake and a smaller Roma population [19]. These findings align with the studies published by Patel et al. and Wang et al. that emphasized the influence of seasonal and regional factors on measles epidemiology [20,21].

Among the adult measles cases reported nationally, 63.3% occurred in unvaccinated individuals, 24.3% were unaware of their vaccine status, and only 5.2% had received the two-dose regimen [9]. Our observations are similar to reports from other European countries, highlighting the need for better record-keeping of the immunization history, vaccination catch-up programs and improved childhood vaccine coverage [22]. Romania reported 78% and 62% vaccination coverage rates for the first and second MMR doses in 2024, which is lower compared with other European countries [6]. The timing of the second dose and catch-up recommendations vary widely between countries, and there is no consensus on the optimal timing in adults [23].

The characteristics of 100 hospitalized adults with measles reflect not only the classical clinical presentation with rash and fever (Table 1) but also showed a high frequency of complications such as hepatic involvement and pneumonitis. These findings confirm the systemic nature of measles in adults, and our results are comparable with studies reporting hepatic (71% to 89%) and pulmonary involvement (40% to 86%) [24,25]. Although previous reports have described high rates of hepatic involvement in association with newly circulating D8 genotype strains, our study cannot establish a direct link between the observed liver impairment and the D8 strain, as viral genotyping was not performed on the nasopharyngeal samples [26,27]. Although complete vaccination did not show significant protection against respiratory complications, the higher frequency of rhabdomyolysis in unvaccinated patients (*p* = 0.003) points to a potential protective effect. No differences related to ethnicity were observed between patients with complications.

The median symptom duration was 5.5 days prior to hospital admission, which indicates a delay in seeking care and could be due to misinterpretation of prodromal symptoms or unawareness of community transmission. In our study, a high proportion of the adult patients did not know the source of infection, and combined with incomplete vaccination, disease susceptibility and the risk for transmission will increase [28].

Among the hospitalized patients in our study, 33% of patients were unvaccinated and 54% were unaware of their status, which equals the national surveillance data. A report from Galati county during the Romanian national 2016–2020 epidemic described that 89.7% of 114 hospitalized adults were unvaccinated and 10.3% had received at least one dose. In contrast to our data, the authors did not differentiate between unvaccinated or unknown immunity status [24]. The proportion of patients with unknown vaccination status in our cohort was similar to a Greek report, where 60.2% of hospitalized adults had unknown vaccination history [25].

Only 49 out of 100 patients completed the survey, and this low response rate may reflect several interrelated factors, including concerns about privacy, fear of judgment or potential consequences, limited trust in medical staff, and the perception that participation offered no direct personal benefit. Ethnic Roma represented 12 out of the 49 confirmed cases (24.5%) within the subset of individuals who agreed to participate in the survey (Table 3). This proportion is higher than previously reported by Turaiche et al. (10.5%) and Fragkou et al. (14.9%) [24,25]. Although in small numbers, our observation indicates a disproportionately high involvement of measles within this community. Low income, low educational level and access to health insurance were significantly different between Romanian and Roma respondents. These socioeconomic disparities are well-documented determinants of health behavior and vaccine hesitancy in marginalized communities [29,30,31].

Survey data reveal different views on MMR vaccination, with 69.4% acknowledging its effectiveness in preventing measles, but concerns about its safety persist. Besides a strong belief in acquired natural immunity, skepticism and fear of autism as an MMR vaccine side-effect was prominent in our study, and Roma ethnicity was significantly associated with this belief. This finding, consistent with prior reports, highlights how misinformation affects marginalized communities, emphasizing the need for targeted education and access to reliable health information [32,33]. However, these observations should be interpreted with caution and not generalized, given the small number of respondents who completed the questionnaire and the inherent limitations of a low-response, cross-sectional sub-analysis.

A critical observation is the reported disparity in income levels; a vast majority of Roma respondents have no income or low incomes compared with the general population, with statistical significance (*p* = 0.040). Lower income, together with limited education, may lead people to trust what they hear from those around them more than formal medical advice, a pattern also noted in other studies on vaccine hesitancy among marginalized groups [34,35].

The analysis also revealed that parental decisions regarding the MMR vaccine were significantly influenced by the number of children in the family. This result resonates with research showing that larger family sizes can sometimes correlate with increased skepticism about vaccine necessity, often due to cumulative concerns over side effects, logistical challenges in healthcare access, and economic constraints [36].

Another dimension of our study worth highlighting is the perspective regarding natural immunity. Approximately one half of respondents believe that acquiring immunity through natural infection might be preferable to vaccination, a belief closely associated with vaccine hesitancy toward the MMR vaccine. This notion, although contrary to abundant evidence confirming the safety and efficacy of the MMR vaccine, persists in part due to historical controversies and the lingering impact of discredited studies linking vaccines to autism. While 85.5% of those who perceived measles as a serious disease support vaccination, the overall mixed views on vaccine safety and efficacy signal a critical need for targeted educational interventions [37].

The fact that only 57.1% of parents reported having vaccinated their children according to the recommended vaccination schedule indicates persistent gaps in knowledge and trust in public health measures. Although there were few participants in our study, this observation calls for a new communication strategy to reach distinct communities like the Roma [38]. Besides vaccine hesitancy, obstacles to attend a general practitioner’s office in rural communities and loss of follow-up are aspects contributing to poor vaccine coverage [6].

Another aspect of understanding the impact of measles within Roma communities is the frequent migration, both domestically and internationally. Measles outbreaks caused by the same D8 genotype strain identified as circulating in Romania have also been reported in several European countries. Thus, several documented measles outbreaks among Roma populations in England and France have been genetically linked to the same D8 genotype strain predominantly circulating in Romania, illustrating the transnational dimension of the problem. For example, the 2019 measles outbreak in Roma settlements in Loire-Atlantique, France, involved a D8 strain that was found to be 100% identical to the strain circulating widely in Romania during the same period. Similarly, multiple outbreaks in English cities such as Birmingham, Leeds, and Liverpool in 2017 and 2018 affected Romanian and Roma Romanian communities, where the D8 strain contributed significantly [39,40].

### Strengths and Limitations

A strength of the study lies in the thorough characterization of 100 adult patients hospitalized with confirmed measles complemented by a dedicated vaccine perception survey and national surveillance statistics. Patient immunization history was discussed in context of national surveillance data from the same time period and our data supports the ongoing national discussion on how to improve vaccination coverage. The study is relevant for health professionals involved in clinical measles assessment and vaccination strategies.

This is a single-center study with a small survey subset and no qualitative data, so the findings reflect hospitalized respondents rather than the full population of adult measles cases. This admission selection bias allows for generalizability to similar patient cohorts, but not to the general adult population. Non-response bias is an important limitation, as 51 of 100 eligible patients did not complete the vaccine perception survey, and no data are available to characterize non-responders; therefore, the direction and magnitude of this bias remain unknown. Nearly one fourth of the hospitalized survey respondent patients were Roma, which is a much higher proportion than in the general Romanian population (last census 2021—3.4% of the total population). Nevertheless, the small Roma subgroup (n = 12) limits the reliability of subgroup analyses; thus, any associations involving Roma participants, including the Roma–autism belief association, should be interpreted as exploratory rather than confirmatory.

## 5. Conclusions

Our study provides insights into the challenges of measles in Romanian adults. National surveillance data reported that unvaccinated individuals, particularly younger adults, were affected by measles across several counties. We observed that hospitalized patients presented classical symptoms along with severe hepatic and respiratory complications causing increased morbidity and prolonged stay. Besides high numbers of incomplete vaccination, a large proportion of patients were unaware of their immunization status. Attitudes toward MMR vaccination varied by ethnicity, with Roma adults exhibiting higher vaccine hesitancy associated with socioeconomic factors such as poor income, low educational level and limited access to healthcare. Targeted and educational programs aimed at reaching marginalized communities are needed to increase MMR vaccination coverage in Romania.

## Figures and Tables

**Table 1:** Patient demographics, comorbidities and clinical characteristics.

*N*	100
Age [mean (SD)]	35 (11.3)
Gender, n	Female 53/100; male 47/100
Environment, n	Urban 67/100; rural 33/100
Vaccination status, n	
Complete (2 doses)	6/100
Incomplete (1 dose)	7/100
Unknown	54/100
Not vaccinated	33/100
Known source of infection, n	Yes 27/100; no 73/100
Days of symptoms before admission [median/range]	5.5 days/1–10 days
Days since onset of skin rash [median/range]	2.4 days/1–7 days
Symptoms and signs	
Rash, n	100/100
Fever, n	99/100
Cough, n	92/100
Koplik spots, n	79/100
Pharyngitis, n	76/100
Nausea/vomiting, n	68/100
Headache, n	48/100
Runny nose, n	36/100
Diarrhea, n	25/100
Dyspnea, n	16/100
Comorbidities	
Hypertension, n	10/100
Diabetes, n	5/100
Pulmonary diseases (asthma/COPD), n	7/100
Malignancies, n	3/100
HIV infection, n	5/100
Chronic viral hepatitis, n	3/100
None, n	65/100

*N*—number of participants, SD—standard deviation, COPD—chronic obstructive pulmonary disease.

**Table 2:** Analysis of the biological parameters, main complications and outcomes of patients hospitalized with measles.

Biological Parameters	
WBC count (cells/mm^3^, mean, SD)	5.690 ± 3666
Neutrophil count at admission (cells/mm^3^, SD)	4.399 ± 1768
Lymphocyte count at admission (cells/mm^3^, SD)	776 ± 247
Thrombocyte count at admission (cells/mm^3^, SD)	172.142 ± 54.085
Neutrophil-to-lymphocyte ratio	10.07 ± 5.89
MDW (mean)	32.35
Fibrinogen (mg/dL) (mean)	433.11 ± 94.90
C-reactive protein (CRP, mean)	45.82
ALT (×ULN, mean)	6.24 ± 7.08
AST (×ULN, mean)	5.35 ± 7.81
CK (U/L), (mean)	369.15 ± 557.39
GGT (U/L), (mean, SD)	279.70 ± 276.38
Complications	
Hepatic involvement *, n	86/100
Pneumonia and/or pneumonitis, n	68/100
Keratoconjunctivitis, n	31/100
Acute respiratory failure, n	21/100
Rhabdomyolysis **, n	11/100
Sinusitis, n	8/100
Acute pancreatitis ***, n	2/100
Treatment and Outcomes	
Length of hospital stay (days, mean, range)	5.57 days/3–15 days
Disease form ****	Mild 11/100 Moderate 68/100 Severe 21/100
Patients receiving antibiotics	Before admission 18/100 During admission 28/100
Bacterial superinfection, n	27/100
Mortality, n	0/100

WBC—white blood cells; MDW—monocyte distribution width; ALT—alanine aminotransferase; AST—aspartate aminotransferase; CK—creatine kinase; GGT—gamma-glutamyl transferase; * alanine aminotransferase (ALT ≥  2 upper limit of normal; ** Serum creatine kinase ≥ 5 normal limits; *** clinical symptoms and high values of lipase; **** mild—no complications, moderate—with pneumonia, severe—with acute respiratory failure.

## Data Availability

The data presented in this study are available on request from the corresponding author.
